# Comorbidity and prognostic indices do not improve the 5-year mortality prediction of components of comprehensive geriatric assessment in hospitalized older patients

**DOI:** 10.1186/1471-2318-14-64

**Published:** 2014-05-15

**Authors:** Nicolás Martínez-Velilla, Koldo Cambra-Contin, Berta Ibáñez-Beroiz

**Affiliations:** 1 Geriatric Department, Complejo Hospitalario de Navarra, Red de Investigación en Servicios Sanitarios en Enfermedades Crónicas (REDISSEC), Irunlarrea 4, Pamplona 31008, Spain; 2 Navarrabiomed-Fundación Miguel Servet, Red de Investigación en Servicios Sanitarios en Enfermedades Crónicas (REDISSEC), Pamplona, Spain

**Keywords:** 5-year mortality, Aged, Comorbidity scores, Multimorbidity, Comprehensive geriatric assessment

## Abstract

**Background:**

Advancing age is associated with increased vulnerability to chronic health problems. Identifying factors that predict oldest-old status is vital for developing effective clinical interventions and public health strategies.

**Methods:**

Observational prospective study of patients aged 75 years and older consecutively admitted to an Acute Geriatric Ward of a tertiary hospital. After a comprehensive geriatric assessment all patients were assessed for five comorbidity indices and two prognostic models. Univariate and multivariate logistic regression models were fitted to assess the association between each score and 5-year mortality. The ability of each score to predict mortality was assessed using the area under the receiver operating characteristic curve.

**Results:**

122 patients were enrolled. All patients were followed up for five years. 90 (74%) of them died during the study period. In the logistic regression analyses, apart from age, cognitive impairment and Barthel Index, three indices were identified as statistically associated with 5-year mortality: the Geriatric Index of Comorbidity and the two prognostic indices. The multivariate model that combined age, sex, cognitive impairment and Barthel showed a good discriminate ability (AUC = 0.79), and it did not improve substantially after adding individually any of the indices.

**Conclusions:**

Some prognostic models and the Geriatric Index of Comorbidity are better than other widely used indices such as the Charlson Index in predicting 5-year mortality in hospitalized older patients, however, none of these indices is superior to some components of comprehensive geriatric assessment.

## Background

Advancing age is associated with increased vulnerability to chronic health problems. Identifying factors that predict oldest-old status is vital for developing effective clinical interventions and public health strategies. In clinical practice many decisions are not fully informed unless the patient´s prognosis is considered. The strongest and most consistent predictors of mortality in older patients include comorbidity and functional status.

There are several important reasons for measuring comorbidity: to correct for confounding factors, to identify the modification of effects, to predict study outcomes or the natural history of the disease, and finally for statistical efficiency [1]. The terms *comorbidity* and *multimorbidity* have been used indistinctly and only in the last few years, a clear distinction between the two terms has been widely accepted [2-4]. Although no “golden standard” for measuring multimorbidity has been established so far, numerous instruments exist, but the issue over which comorbidity index is the best for research purposes is still unclear. A range of morbidity scores have been used, of which the Charlson Index (ChI) [5] is the most well known, but evidence for the limitations of this index in older patients is growing [6]. There have been several reviews of prognostic and comorbidity indices for older adults [1,4,7-9]. However, although these indices try to help in the clinical decision-making process, there is still insufficient evidence at this time to recommend the wide-spread use of prognostic indices in clinical practice. Surprisingly, even the more-common models developed some 25 years ago have not been frequently validated in an older subgroup. The applicability of these models in this increasingly important population merits special attention.

Our previous study showed that comprehensive geriatric assessment (CGA) is a better 1-year mortality predictor than several indices and factors [10], and the objective of the present study is to evaluate the long-term prognostic ability of these factors and indices in order to check their eventual role if added to functional and cognitive measures.

## Methods

This is an observational prospective study of patients aged 75 years and older consecutively admitted to an Acute Geriatric Ward of a tertiary hospital in Spain. After a comprehensive geriatric assessment of all the relevant clinical variables. This research was carried out in compliance with the Helsinki Declaration, and informed written consent was obtained from the patient or the next of kin to enter in the study. The assessment included clinical and demographic information, as well as an assessment of dependence in daily activities by means of the Barthel Index [11]. All patients underwent a mental function evaluation using the Spanish version of the Folstein Mini-Mental State Examination [12], with scores ranging from 0 (poor) to 35 (excellent). The same clinical interviewer assessed the following comorbidity and prognostic indices:

### Prognostic indices

The *Burden of Illness Score for Elderly Persons* (BISEP) by Inouye et al. [13] updated previous indices which had been developed by the same group by adding functional and laboratory data to the diagnoses of administrative data in order to estimate 1-year mortality. Participants were drawn from a prospective study of individuals aged 70 years and older who were hospitalized at Yale-New Haven Hospital. The study was validated in a sample of 1246 participants from 27 Connecticut hospitals who were 65 years and older with a principal discharge diagnosis of pneumonia. The investigators demonstrated improvement in the C statistic with the addition of laboratory and functional cognitive measures to administrative data.

The *Prognostic Index* (PI) proposed by Walter et al., [14] was developed to predict 1-year mortality for elderly individuals after hospital discharge using secondary data from a study of patients aged 70 years and older who were hospitalized at the University of Hospitals Cleveland and the Akron City Hospital. Rozzini subsequently externally validated the index´s performance predicting 6-month mortality in a retrospective analysis of 840 consecutively admitted participants to a hospital in Italy and found monotonic increases in mortality for each predicted risk level.

### Comorbidity indices

The *Charlson Index*[5] (ChI) is the most extensively studied comorbidity index. It was originally developed to predict 1-year mortality in 604 patients [5]. It features 19 conditions with assigned weights from 1 to 6, and a total score is obtained by adding up the weights of all present comorbidity illnesses. The diseases were selected on the basis of the degree of their association with mortality. Although the maximum score is 37, patients rarely score above 3 (cancer patients).

The *Index of Coexistent Disease* (ICED) was designed by Greenfield [15] to explain variations in patient health status at hospital admission and discharge, and it is based on the presence and severity of 19 medical conditions and 11 physical impairments, using two subscales: the Individual Disease Severity (IDS) and the Functional Severity (FS). The IDS grades each condition on a 0-4 scale. Scores are based on an explicit list of symptoms, signs, and laboratory tests. Using pre-specified grouping rules, the IDS and FS scores can be combined to create a single score from 0 to 3. We used the total score, which was categorized into quartiles.

The *Diagnosis Count* (DC) is a simple and universally available comorbidity index consisting of a numeric sum of all secondary diagnoses and has been demonstrated to be predictive of overall mortality and hospital resource utilization [16,17]. Although this method seems to be quite straightforward, substantial differences exist with regard to the definition used to consider a condition as comorbid.

The *Cumulative Illness Rating Scale* (CIRS) [18] rates 14 body systems on a five-point severity scale. It was adapted to be used in geriatric populations (CIRS-G) (CIRS geriatric),for which guidelines to enhance reliability have been formulated [19]. Each system is scored as follows: 1(none)-no impairment to that organ or system; 2(mild)-impairment does not interfere with normal activity, treatment may or may not be required, prognosis is excellent; 3(moderate)- impairment interferes with normal activity, treatment is needed, prognosis is good; 4 (severe)- impairment is disabling, treatment is urgently needed, prognosis is guarded; 5 (extremely severe)- impairment is life threatening, treatment is urgent or of no avail, poor prognosis.

The *Geriatric index of comorbidity* (GIC) [20] classifies patients into 4 classes of increasing somatic comorbidity. The GIC was defined based on information coming from two domains, number of diseases and severity of diseases as measured by Greenfield´s IDS. Class I includes patients with one or more conditions with IDS = 1 or lower. Class II includes patients with one or more conditions with IDS = 2. Class III includes patients with one condition with IDS = 3, other conditions having IDS = 2 or lower. Class IV includes patients with IDS = 3 or one or more conditions with IDS = 4.

### Outcome measure

All patients were followed up for five years and assessed in the outpatients clinic or via a telephone interview, and the information was then checked with the computerized clinical history. Then we examined the performance of each comorbidity measure with respect to predicting 5-year mortality after hospital discharge.

### Statistical analysis

Demographic and clinical characteristics of the patients were summarized using descriptive statistics such as frequency and percentages for categorical variables and mean and standard deviation for continuous ones. Scores were categorized into quartiles to facilitate comparisons between indices. Univariate logistic regression models were fitted to assess the association between each score and 5-years mortality, which provided Odds Ratios (OR) with their 95% confidence intervals (CI) for the scores’ quartiles. Logistic regression analysis models also provided the pseudo R^2^, which was used to compare the percentage of variance explained by each model. The ability of each score to predict mortality was assessed using the area under the receiver operating characteristic curve (AUC), and a value of 0.7 or higher for the AUC was accepted as clinically relevant. Finally, to assess the contribution of each index over components of comprehensive geriatric assessment (age, function, and cognition), a multivariate logistic regression model with the covariates was first fitted, and its AUC was compared with the AUCs obtained by adding independently each one of the scores. Statistical analysis was performed using SPSS for Windows 21.0 (SPSS, Inc., Chicago, IL).

The study was performed in accordance with the Declaration of Helsinki. Informed consent to participate in the study was obtained from all participants or proxies (if the patient had cognitive impairment).

## Results

122 patients were enrolled. Mean age was 85.4 (5.4 SD), 56.6% were women, 21% were living in nursing homes, 48.2% had at least mild cognitive impairment (12.3% severe) and 62.3% of them were taking 6 or more drugs. 51.6% of the patients had a Barthel Index lower than 75 (mean 65.2 SD 34.41). The main diagnoses on admission were acute cardiac and respiratory exacerbations. All patients were followed up during the five years. 90 (74%) of them died during the study period (22% within the first month and 50% over the first 4 months). The results of the univariate logistic regression models are given in the left- hand side of Table 1, which shows that apart from age (p = 0.001) and, marginally, Barthel (p = 0.056), the indices GIC (p = 0.012), BISEP (p = 0.01) and PI (p = 0.005) were statistically associated with 5-year mortality. The risk gradient across quartiles for these three scores is remarkable, as can be picked out from the estimates of the Odds Ratios. The Pseudo R^2^ statistics was also highest for the univariate models with each of these three indexes (R^2^ = 16.1%, 17.3% and 20.9% respectively). In the same direction, they showed the best predictive ability according to the AUC (0.66, 0.73 and 0.72 respectively), the last two show good predictive ability (≥0.70). The multivariate model that combined age, sex, cognitive impairment, and Barthel showed a good discriminate ability (AUC = 0.79), and results did not improve substantially after adding individually any of the indices, as can be seen on the right-hand side of Table 1.

**Table 1:** Logistic regression analysis predicting 5-year mortality

		**Univariate logistic regression**				**Multivariate**
		**OR (IC**_**95%**_**)**	**p**	**R**^**2**^**(%)**	**AUC**	**AUC**^(*)^
***Covariates of adjustment***						
**Age**	(Continuous)	1.17(1.06,1.28)	0.001	14.6	0.70(0.59,0.81)	
**Sex**	Female	Reference				
	Male	1.17(0.52,2.65)	0.708	0.2	0.52(0.40,0.64)	0.79(0.71,0.88)
**Cognitive**	No	Reference				
**Impairment**	Mild	2.02(0.66,6.24)				
	Mod/severe	7.58(2.10,27.4)	0.007	15.3	0.69(0.59,0.79)	
**Barthel**	4	Reference	0.056	11.2	0.72(0.62,0.82)	
	3	2.14(0.70,6.58)				
	2	2.18(0.55,8.68)				
	1	7.14(1.54,33.15)				
***Comorbidity indices***						
**ChI**	1	Reference				
	2	1.92(0.66,5.61)	0.144	7.0	0.64(0.53,0.75)	0.81(0.73,0.89)
	3	2.30(0.67,7.90)				
	4	4.03(1.06,15.31)				
**DC**	1	Reference				
	2	5.76(1.17,28.24)	0.106	8.1	0.58(0.45,0.70)	0.80(0.72,0.88)
	3	2.38(0.84,6.75)				
	4	1.92(0.66,5.56)				
**CIRS**	1	Reference				
	2	1.23(0.40,3.78)	0.601	2.4	0.54(0.42,0.66)	0.80(0.71,0.88)
	3	0.97(0.34,2.80)				
	4	2.25(0.60,8.46)				
**ICED**	1	Reference				
	2	0.70(0.21,2.34)	0.344	4.5	0.56(0.45,0.67)	0.79(0.71,0.87)
	3	0.75(0.26,2.12)				
	4	2.17(0.58,8.20)				
**GIC**	1	Reference				
	2	0.50(0.15,1.67)	0.012	16.1	0.66(0.56,0.76)	0.79(0.71,0.88)
	3	1.04(0.27,4.01)				
	4	4.62(0.96,22.09)				
***Prognostic indices***						
**BISEP**	1	Reference				
	2	1.41(0.52,3.85)	0.010	17.2	0.73(0.63,0.82)	0.81(0.73,0.89)
	3	6.92(1.29,37.05)				
	4	10.0(1.91,52.47)				
**PI**	1	Reference				
	2	3.10(0.83,11.66)	0.005	20.9	0.72(0.62,0.83)	0.80(0.71,0.88)
	3	5.85(1.52,22.57)				
	4	18.00(4.01,80.71)				

BISEP: Burden of Illness Score for Elderly Persons; ChI: Charlson Index; CIRS: Cumulative Illness Rating Scale; DC: Disease Count; ICED: Index of Coexistent Disease; GIC: Geriatric Index of Comorbidity; PI: Prognostic Index *Quartile ranges do not aply to ICED and GIC, as these indices are predefined into 4 classes

(*) The multivariate AUC for the “covariates of adjustment” refers to the model including only age, sex, Barthel and cog impairment, whereas the multivariate AUC given for each index is the one obtained from the multivariate model including the formers and each one of the indices.

## Discussion

This study explores the relationship between several comorbidity and prognostic indices and mortality in older hospitalized patients. It identifies which of these indices are more appropriate in the older hospitalized patients and features that components from the comprehensive geriatric assessment (function and cognition) are the cornerstone for prognostic decisions. Our work confirms previous findings that have shown that the Charlson index, used alone, is not the best index in such a complex population [21-25], and adds an innovative approach comparing comorbidity indices and prognostic models, showing that BISEP and PI are useful prognostic tools. The persistence of the same factors to predict 1 and 5-year mortality empower the solidity of our model [10].

Currently, there is no accepted standardized method for measuring and quantifying the prognostic value of comorbid conditions in hospitalized elderly patients with acute disease. Because of competing chronic conditions and diminished life expectancy, careful considerations of prognosis is particularly important for clinical decision making in older patients. It seems that GIC is more suitable in older hospitalized patients as Zekry et al demonstrated [21-23]. They also compared 6 comorbidity indices for one and five-year mortality prediction and found that GIC and CIRS-G were the best predictors. Rozzini [20] did the same in Geriatric Evaluation and Rehabilitation Units for subacute and disabled patients with four indices. They found that GIC had the greatest concurrent validity with disability and was the best predictor of mortality. On the other hand, Di Bari et al., [26] found that DC, ChI and ICED performed better than GIC. This was probably because their population was younger (mean age was 74 ± 0.3) and less complex than ours and that included in the study of Zekry et al. [21] The GIC captures a significant part of the excess disability due to the co-occurrence of diseases but its hierarchical construction does not allow for the discrimination of degrees of comorbidity conditions whose severity is lower than that of the most severe conditions. It can help clinicians to discriminate and adjust basal status in the different study populations so that a stratification for the analysis can be performed and reliable conclusions be drawn afterwards. None of the C statistics for the included indices were higher than 0.90, similarly to previous studies that showed C statistics between 0.662 and 0.77. This discriminatory ability is consistent with other indices that commonly drive clinical decisions, such as the LACE index (C statistic, 0.684) [27], the TIMI risk score (C statistic, 0.65) [28], the CHADS2 index ( C statistic, 0.68-0.72) [29] or the Framingham risk score (C statistic, 0.63-0.83) [30].

The results of the current study have also shown that none of the indices improves the prognostic approach of the CGA in predicting survival and adds to the growing evidence that CGA is a better predictor than comorbid conditions, and is able to help to improve care planning, and overall better quality of care. [31-34] Other authors have shown that disability, more than multimorbidity, exerts an important influence on mortality, independently of age and other clinical functional variables [35]. Furthermore, among hospitalized elderly subjects, functional impairment rather than comorbidity and indices of disease burden have been recognized as the strongest predictors of outcomes [36-38]. The Barthel Index could therefore be more useful than other indexes when considering an adequate use of healthcare services [24]. Cognitive impairment is the other pillar of this model, and it has been validated as an excellent predictor of adverse outcomes.

We have to analyze these conclusions in a comprehensive geriatric context. Even if function and cognition are the main long term mortality predictors, we should always consider other areas like comorbidity, social support or age. As a matter of fact, function and mental status could be recognized as the background defining elderly health status and help to stratify a spectrum of alternative therapeutic approaches, from life saving to palliative and symptomatic care. All these issues are complementary and not mutually exclusive.

The main strength of this study is the prospective comprehensive assessment of several indices and prognostic models of mortality. As far as we know, this is the first prospective study comparing at the same time, comorbidity indices, prognostic models and CGA for the prediction of 5-year mortality. Previous studies have used only few or just one index and have been done in a community-dwelling population, and only a few of them present the coefficient of determination (R^2^) of their model [9,20,39]. Our study is difficult to generalize because the data are obtained in a small sample of hospitalized older patients with a high burden of comorbidities, disability and cognitive impairment.

## Conclusions

In conclusion, BISEP, PI and GIC indices are better than other widely used indices such as ChI in predicting 5-years mortality in hospitalized older patients, but none of these indices are superior to some components of CGA. The evidence that function and cognition have a greater effect than multimorbidity on mortality prediction suggests moving from a disease-centered model for frail older patients to an integrative cognitive and functional management.

## Abbreviations

BISEP: Burden of illness score for elderly persons; CGA: Comprehensive geriatric assessment; CIRS: Cumulative illness rating scale; ChI: Charlson index; DC: Diagnosis count; GIC: Geriatric index of comorbidity; ICED: Index of coexistent disease; IDS: Individual disease severity; PI: Prognostic index.

## Competing interest

The authors declare that they have no competing interests.

## Authors’ contributions

NM carried out the conception, design and acquisition of data, and wrote the manuscript. KC carried out the statistical analysis and helped to draft the manuscript. BI carried out the statistical analysis and helped to draft the manuscript. All authors read and approved the final manuscript.

## Pre-publication history

The pre-publication history for this paper can be accessed here:

http://www.biomedcentral.com/1471-2318/14/64/prepub
